# DFT-Assisted Spectroscopic Studies on the Coordination of Small Ligands to Palladium: From Isolated Ions to Nanoparticles

**DOI:** 10.1021/acs.jpcc.9b09791

**Published:** 2020-01-27

**Authors:** Sebastiano Campisi, Cameron Beevers, Ali Nasrallah, C. Richard A. Catlow, Carine e. Chan-Thaw, Maela Manzoli, Nikolaos Dimitratos, David J. Willock, Alberto Roldan, Alberto Villa

**Affiliations:** † Dipartimento di Chimica, Università degli Studi di Milano, Via Golgi 19, I-20133 Milano, Italy; ‡ Cardiff Catalysis Institute, School of Chemistry, Cardiff University, Main Building, Park Place, CF10 3AT Cardiff, U.K.; § Department of Drug Science and Technology and NIS—Centre for Nanostructured Interfaces and Surfaces, University of Turin, Via P. Giuria 9, 10125 Turin, Italy; ∥ Dipartimento di Chimica Industriale e dei Materiali, Alma Mater Studiorum Università di Bologna, Viale Risorgimento 4, 40136 Bologna, Italy

## Abstract

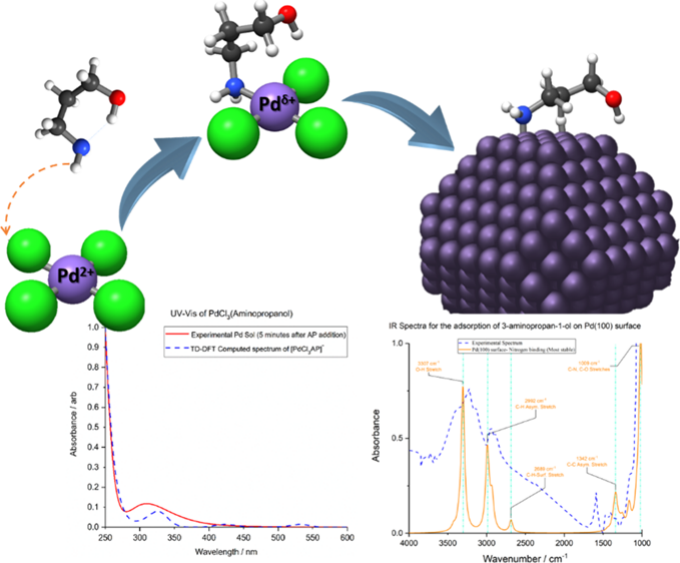

A combination of experimental spectroscopies (UV–vis and Fourier-transform infrared) and computational modeling was used to investigate the coordination of small ligands (aminopropanol and propanediol) to Pd species during the metal nanoparticle formation process. Differences emerged between O- (propanediol) and N-containing (aminopropanol) ligands. In particular, a strong interaction between the NH amino group and Pd^2+^ ions could be inferred on the basis of spectroscopic evidences, which was corroborated by theoretical simulations, which confirmed the preferential coordination of aminopropanol through the NH group. This interaction seems to potentially cause the aminopropanol ligand to control the particle shape through a selective blocking of Pd(100) facets, which promote the growth on the Pd(111) facets.

## 1 Introduction

In the last decades, metal nanoparticles (MNPs) have been used increasingly as key components for applications in several fields such as energy conversion and storage, biomedicine and life science, electronics, information technology, and catalysis, thanks to their unique and fascinating properties.^1−4^ Most of the physicochemical properties of MNPs (e.g., optical, catalytic, magnetic, and electronic properties) are highly dependent on a set of structural and morphological parameters, including composition, particle size, shape and exposure of facets, crystal structure, surface modification, and environment. Despite this strong relationship between their structure and function, the practical applications and the performances of MNPs are still limited by the lack of a clear predictability of the synthesis outcome in terms of size and morphological dispersion.^5,6^

Indeed, despite extensive studies and significant advances, the development of synthetic routes able to produce MNPs with an enhanced degree of compositional, dimensional, morphological, and structural control still remains an open challenge. This difficulty arises, in part, from the significant number of factors affecting solution-based methods which need to be considered and tuned in order to achieve the rational design of size- and shape-controlled MNPs syntheses. The metal reduction potential, the nature and concentration of precursors, the reducing agent, the solvent, the temperature, and the mass transfer phenomena have a considerable impact on the mechanisms of nucleation and growth.^7−10^ In addition, capping agents are commonly used in solution-phase synthesis to stabilize MNPs and to prevent their aggregation. The capping agents are often selected from various types of molecules, such as thiols, amines, halides, carboxylic acids, phosphines, polymers, and surfactants.^11−13^ Capping agents can play multiple roles including providing colloidal stabilization and acting as structure-directing agents.^7,11,14−19^ Furthermore, as with conventional ligands, the capping agents can coordinate metal centers during different stages from metal precursors to MNPs. During the synthesis process, the metal precursor is decomposed and reduced into metal atoms, which then aggregate and evolve toward the formation of clusters and then nanoparticles (NPs). The role of the capping agents in the overall thermodynamics and kinetics of the nucleation-growth processes derives from the different interactions with metal ions, MNPs, and several intermediates, as shown by many experimental studies.^6,7,11,12,17,20,21^ The concentration ratio of the capping agent and metal has been demonstrated to affect the final sizes of alkanethiolate-capped Au NPs.^22^ The interaction strength influences the particle size as reported by Karim et al. for Pd NPs synthesized in the presence of oleylamine (weak capping agent) or trioctylphosphine (TOP) (strong capping agent).^23^ The capping agent can also induce significant variations in the electrochemical reduction potential of metal ions, as suggested by the studies by Biacchi and Schaak concerning the effect of different polyols on the reduction of Rh salts.^24^

More recently, by combining in situ small-angle X-ray scattering (SAXS) and kinetic modeling, Mozaffari et al. investigated in detail the mechanisms of Pd NPs nucleation and growth.^25^ The study demonstrated that in different solvents (pyridine and toluene), the capping agents, acetate, and TOP, can exert a kinetic control on both the nucleation and growth rates, which was achieved by examining the concentration of the kinetically active metal precursor and the number of free surface sites on the respective NPs’ facets.

The capping agent not only influences the formation of MNPs but can also irreversibly adsorb onto their surfaces, affecting their performance and utility, which can drastically reduce the activity of heterogeneous catalysts.^26−28^ It has been demonstrated that adsorbed capping agent molecules create an interphase, where diffusional, steric, and electronic effects can control and modify the overall activity and selectivity of catalytic reactions.^27^ The reaction pathway is often governed by the preferential interactions between capping agent molecules and specific active sites (facets, edges, corners, and defects) on the MNP surfaces.^28^

For these reasons, a significant amount of research has been devoted recently to investigate the connection between metal–capping agent interactions and the formation mechanisms of MNPs in solution, as well as the nature and role of the local ligand environment of metal species in solution. Several studies have been reported using advanced characterization techniques (e.g., SAXS, liquid cell transmission electron microscopy, and extended X-ray absorption fine structure) to provide a molecular-level understanding of the roles of capping agents during the formation of MNPs.^7,11,17,19−21,29−43^ Here, we employ a combination of UV–visible (UV–vis) and Fourier transform infrared (FT-IR) spectroscopies and density functional theory (DFT) modeling to investigate the coordination of small ligands (diols and amino alcohols) to Pd^2+^ ions and Pd NPs. The complementary use of conventional spectroscopic techniques and theoretical modeling has already been demonstrated to be a powerful tool in investigating metal–ligand interactions in Au and Ag NPs.^34−36,40,44^ The pH dependence of interaction strength and conformation of thiolate and thione molecules at the surface of Au NPs has been studied by means of surface-enhanced Raman spectroscopy (SERS) combined with DFT modeling by Ansar et al.^36^ The synthesis of amidine-stabilized Ag NPs via hydrogenolysis of silver amidinate in the presence of hexadecylamine was explored by Cure et al. through nuclear magnetic resonance and SERS spectroscopies in combination with DFT simulations devoted to unraveling the coordination of ligands to Ag NPs.^40^ Singh et al. monitored the synthesis of curcumin-capped Au NPs by in situ UV–vis Spectroscopy, and experimental results were correlated with DFT calculations exploring the formation of several complexes of curcumin with Au^3+^ ions in various conformational isomeric forms.^35^ In this study, we focused on Pd NPs, which were selected as a model metal system because of their prevalence and relevance to heterogenous catalysis. DFT-derived spectra were used to interpret the experimental results in detail. Our results illustrate the DFT-assisted spectroscopic approach to describe the ligand coordination to NP’s facets. The species present in the experimental reaction solutions have been examined through comparison of experimental with computed spectra.

## 2 Experimental Section

### 2.1 Synthesis of Pd NPs

Solid Na_2_PdCl_4_ (0.094 mmol of Pd) and aminopropanol or propanediol water solution (1 wt %) (Pd/capping agent 1:100 weight ratio) were added to 100 mL of H_2_O. After 3 min, NaBH_4_ (Pd/NaBH_4_ = 1/8 mol/mol) solution was added to the yellow-brown solution under vigorous magnetic stirring. A brown Pd(0) sol was immediately formed.

### 2.2 Spectroscopic Studies

UV–vis spectra of sols were recorded using a PerkinElmer λ25 spectrophotometer in H_2_O between 190 and 1200 nm using a quartz cuvette. The samples were loaded into a rectangular quartz cuvette of 1 cm width and 3 cm height. The sample measurement was made with respect to a reference scan of the solvent (i.e., distilled water). Spectra were recorded after 10, 60, and 300 s from the addition of the capping agent.

FT-IR experiments were performed with cells allowing spectrum scanning on the liquid samples at room temperature. The FT-IR spectra were recorded using a PerkinElmer 2000 spectrometer (equipped with a cryogenic HgCdTe (MCT) detector). The spectra were acquired in the 4000–1000 cm^–1^ range with a 2 cm^–1^ resolution. To collect the FT-IR spectra of our aqueous solutions, we employed a commercial demountable transmission cell equipped with CaF_2_ windows (shown in Figure SI-1 of the Supporting Information). The solution is dropped onto a CaF_2_ window and sandwiched with another equal window such that no gas bubbles are trapped. This procedure implies that the amount of sample is very low, and the contribution of the solvent is negligible under these conditions.

The measured solution sample forms a thin liquid film between the two windows, which is typically less than 0.01 mm thick. It is not possible to measure the optical path, being that of the liquid film. Indeed, as the thickness is not constant from measurement to measurement, this type of cell is unsuitable for quantitative analysis.

### 2.3 Computational Details

This project uses DFT as incorporated within the ORCA^45^ and VASP (Vienna Ab initio Software Package)^46−49^ simulation codes to model precursor molecules and experimentally formed NPs, respectively, the latter of which is modeled as extended periodic surfaces. The Perdew–Burke–Ernzerhof (PBE) exchange–correlation functional^50,51^ was employed to account for the exchange and correlation effects on valence electrons with the projector augmented-wave method used to represent atomic core states.^52,53^ To ensure consistency between periodic and nonperiodic simulations, extended tests were carried out using PBE and Becke–Perdew-86 functional (BP86). These consistency tests have shown the PBE functional to be a reasonable compromise between cost and accuracy for the second-row transition metals modeled using both periodic and atom-centered DFT methodologies.^54^ Dispersion effects were included in both atom-centered and plane-wave models using Grimme’s empirical DFT-D3 model.^55^ For nonperiodic, atom-centered models, DFT-D3BJ (DFT-D3 with Becke–Johnson damping) was used to prevent artificial short-range repulsive interactions.^55−58^

Dipole correction along the *Z*-direction of the periodic slab model was applied when necessary. A Monkhorst–Pack grid was used to sample the Brillouin zone.^59^ For optimization calculations, the number of *k*-points used was 7 × 7 × 7 for the optimization of the fcc unit cell bulk structure, and 7 × 7 × 1 for the surfaces. For the adsorption calculations on the (111) surface, a 5 atomic layer-thick slab with a *p*(4 × 4) surface supercell (80 atoms) was employed. For the (100) surface, a *c*(3 × 3) surface slab was constructed, again 5 layers thick (90 atoms). During optimization calculations, the top two layers of the slabs were relaxed and the lower 3 layers fixed at their optimized bulk positions. A 3 × 3 × 1 *k*-points sampling was used for all surface calculations. The periodically repeated slabs were separated by a 20 Å vacuum layer along the *Z* direction, which is enough to avoid any spurious interaction with periodically replicated images. A kinetic energy of the plane waves was set to 400 eV ensuring no Pulay stress. The convergence criterion was set such that the calculations converge when the forces are less than 0.02 eV Å^–1^ for adsorption calculations, 0.001 eV Å^–1^ for the bulk, and 0.01 eV Å^–1^ for surface optimization calculations. The adsorption energy was computed using eq 1.
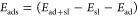
1where *E*_ad+sl_ is the energy of the adsorbate adsorbed on the slab, *E*_sl_ is the energy of the naked slab, and *E*_ad_ is the energy of the adsorbate in the gas phase, that is, in a cell large enough to avoid intermolecular interactions.

The ORCA implementation of Karlsruhe quadruple zeta with valence and polarization function basis set (def2-QZVP), the auxiliary Weigend basis set (def2/J), and Stuttgart–Dresden effective core potentials was used for all nonperiodic atom-centered DFT calculations.^60−62^ The convergence criteria for these calculations were an energy change of 2.72 × 10^–5^ eV with a maximum gradient of 5.14 × 10^–3^ eV Å^–1^ and a maximum displacement of 5.29 × 10^–4^ Å. Analytical frequency calculations were also performed to optimize and confirm the geometry of the aminopropanol ligand molecular precursor.

The species present in the reaction solutions have been examined through comparison of experimental with computed spectra. Simulated UV–vis spectra were calculated using the simplified Tamm–Dancoff approximation of time-dependent DFT (sTDA-DFT), which has been shown to give good agreement with TD-DFT for the electronic transition energies, although it is known that intensities calculated using this method are less reliable.^63^ The calculation efficiency of sTDA-DFT has also been increased by employment of the RIJCOSX approximation of the Coulomb and exchange integrals. Structures showing sTDA-DFT transitions consistent with the wavelength of experimentally observed bands were also calculated using the more demanding TD-DFT with the RIJCOSX integral approximation. TD-DFT spectra were examined using the Multiwfn software package, which applies Gaussian curve broadening.^64^ Calculated excitations and orbital compositions were determined using the Mulliken method.^65^ Localized orbital centroid analysis was also undertaken in order to examine the ligand bonding and oxidation state of the Pd^2+^ ions in the NP precursor molecule using the methodology of Vidossich and Lledós.^66^ This utilized the ORCA software package’s implementation of the Pipek–Mezey population-localisation methodology to derive localized orbitals from the DFT-calculated electronic structure.^67,68^ The gas-phase energy changes of reaction, Δ*E*_r_, were calculated using eq 2.
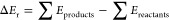
2

## 3 Results and Discussion

The coordination of the capping agent to Pd^2+^ ions and Pd NPs was investigated by UV–vis and FT-IR spectroscopies. 3-Aminopropanol (AP) and 1,3-propanediol (PD) were studied as model capping agents for the Pd sol. These molecules were selected because their molecular structures resemble the repeating units of several polymers commonly used as capping agents for Pd NPs (e.g., poly vinyl alcohol, poly ethylene glycol, and poly vinyl pyrrolidone).

**Figure 1:**
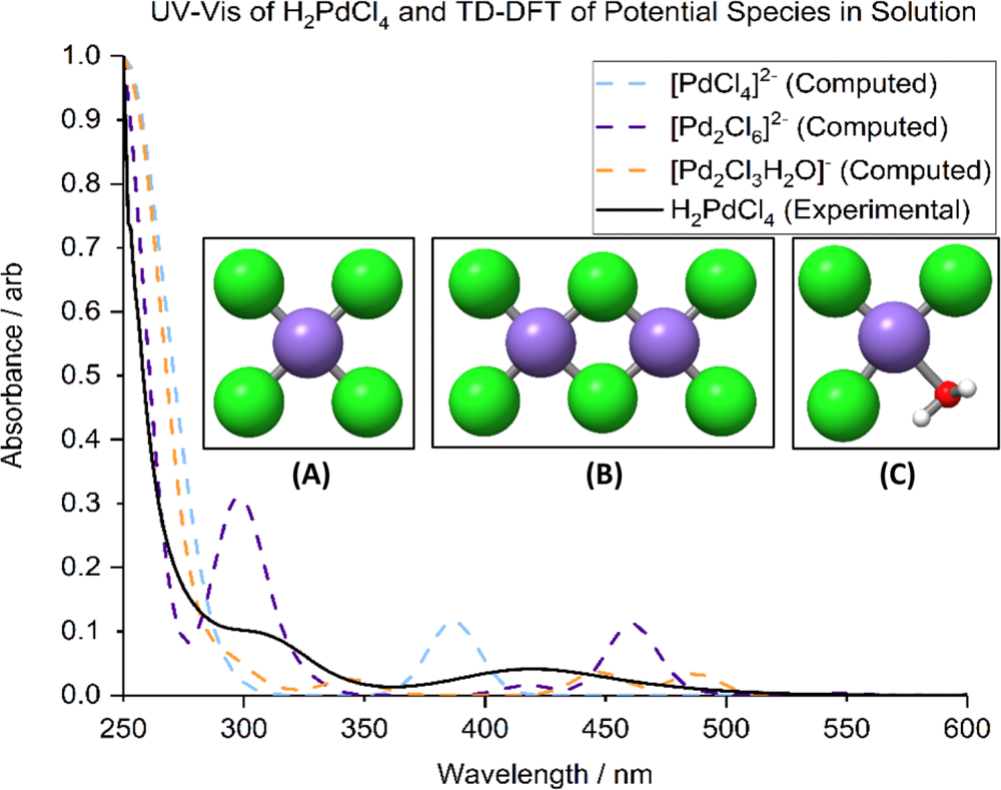
Experimental UV–vis spectra of H_2_PdCl_4_ in solution at pH 3 (black), computed UV–vis spectra of [PdCl_4_]^2–^, [Pd_2_Cl_6_]^2–^, and [PdCl_3_H_2_O]^−^. Inset: Structures of PdCl_4_^2–^, (A); Pd_2_Cl_6_^2–^, (B); and [PdCl_3_(H_2_O)]^−^, (C). Color code: Pd: cyan; Cl: green; O: red; and H: light gray.

**Figure 2:**
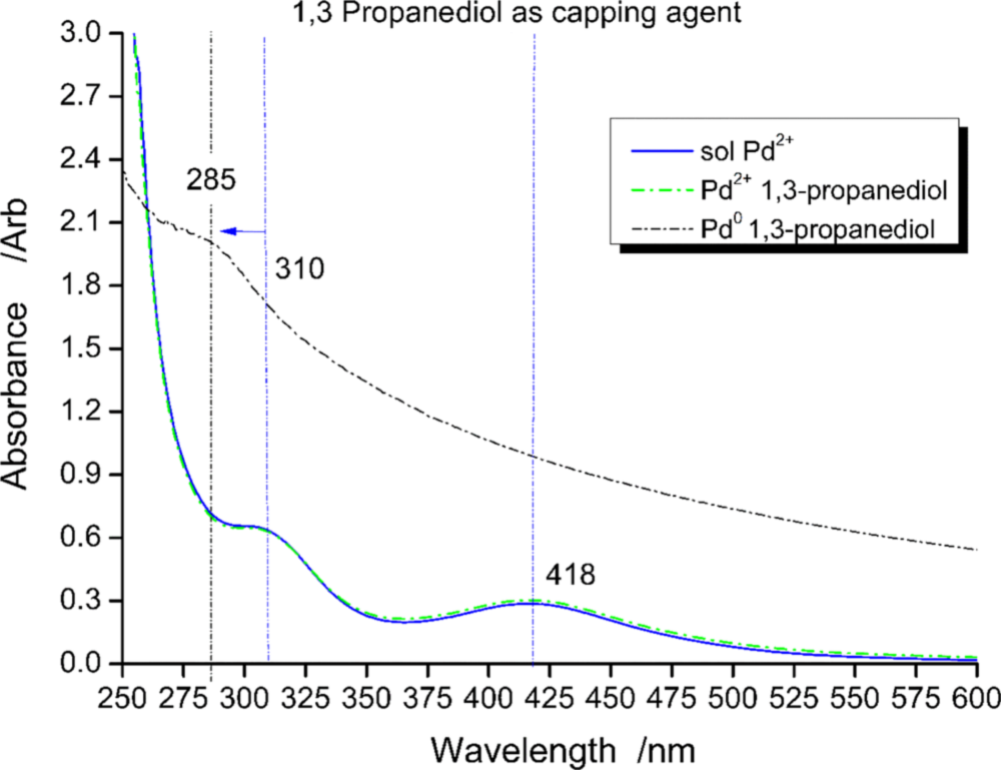
UV–vis spectra of H_2_PdCl_4_ in solution (blue) in the presence of the capping agent PD (green), Pd with PD reduced (black).

**Figure 3:**
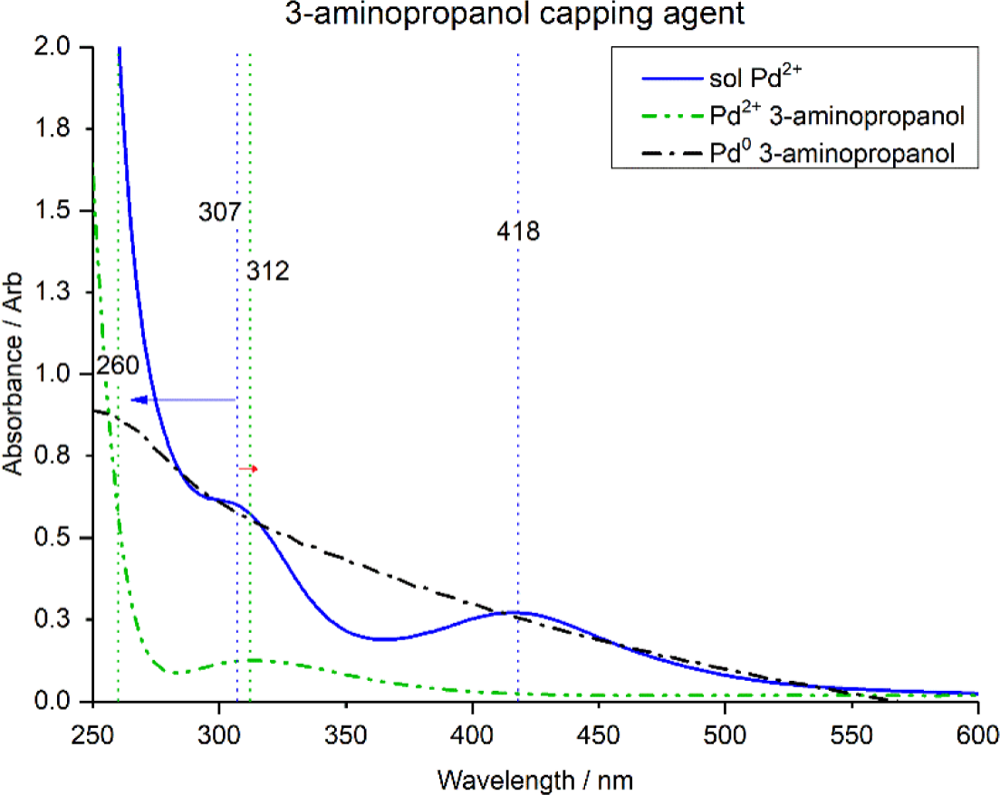
UV–vis spectra of H_2_PdCl_4_ in solution (blue), in the presence of the capping agent AP (green), Pd with AP reduced (black).

The coordination of the capping agent molecules to Pd^2+^ ions was monitored in real time by UV–vis spectroscopy under typical synthetic environments for the generation of the Pd sol. The starting H_2_PdCl_4_ salt in solution (the black line in Figures 1–3, which show the observed and calculated spectra) is characterized by UV–vis bands at 310 and 418 nm. These bands could be associated to ligand to metal charge-transfer (CT) and d–d electronic transitions. Typically, bands originated from d–d transitions are much less intense and more resolved than CT transitions. On the other hand, the identification of the Pd species responsible for these spectral features is no trivial matter. As reported by Elding, the predominant species found in acidic media are PdCl_4_^2–^ and [PdCl_3_(H_2_O)]^−^.^999^ Spectroscopic evidences of the formation of these species were obtained also using solid PdCl_2_ as the precursor by Freund et al., who collected UV–vis spectra of solutions depending on the pH of the media.^70^ At a pH of 1.3, d–d and CT transitions at 280 nm/475 nm for PdCl_4_^2–^ and 320 nm/430 nm for [PdCl_3_(H_2_O)]^−^, respectively, were observed. At a pH of 10, a spectrum showing increased background absorption was observed along with an increase at short wavelengths with a feature centered at 270 nm. In addition, Grogan and Nakamoto reported the formation of dimeric species. Pd_2_Cl_4_(EthO)_2_^2–^ which can be described as a Pd analogue to the Pt salt Zeise’s dimer.^71^

To identify the contribution of each of these species to the experimental spectra TD-DFT simulations were carried out to model UV–vis absorption spectra of PdCl_4_^2–^, [PdCl_3_(H_2_O)]^−^, and Pd_2_Cl_6_^2–^ species. TD-DFT-computed UV–vis spectra of PdCl_4_^2–^ and Pd_2_Cl_6_^2–^ (green and blue dotted curves in Figure 1, respectively) are in good agreement with the observed experimental spectrum (the black curve in Figure 1). The computed data for the chlorine bridged dimer indicate that the characteristic 420 nm band in the experimental spectra is due to Cl p–Pd s transition. However, the breadth of this band is likely due to the contributions of 470 nm transition, indicating a predominantly Pd d to Pd p excitation involving both palladium ions of the dimer, and a 388 nm transition observed in the computed spectrum of PdCl_4_^2–^, attributable to a predominantly p–p excitation from chlorine to palladium. Therefore, the experimental band centered at 418 nm could include all the contributions predicted by the model systems.

The inherently large uncertainty in the relative intensities of bands obtained using the TD-DFT methodology means that the position of the peaks is used to analyze the spectra.^63^ The broad band present at 425 nm is probably composed of a combination of peaks caused by contributions of the three computationally modeled structures. Furthermore, the shoulder observed in the experimental spectrum at 310 nm is consistent with the computed spectrum of the dimer shown in Figure 1B. The agreement of these computed excitations with experimental spectra suggests that the sol is composed of an equilibrium mixture of Pd_2_Cl_6_^2–^, PdCl_4_^2–^, and [PdCl_3_(H_2_O)]^−^. The existence of these species is also supported by the calculation of the energies of reaction for the formation of Pd_2_Cl_6_^2–^ and [PdCl_3_(H_2_O)]^−^ from PdCl_4_^2–^, shown in Table 1. These values indicate that the formation of the dimer and the water-containing complex are both exothermic processes with the dimer being the more energetically favorable.

**Table 1:** Energy Change of Reaction (Δ*E*_r_) for the Formation of Pd_2_Cl_6_^2–^ and [PdCl_3_H_2_O]^−^ from PdCl_4_

products	Δ*E*_r_/kJ mol^–1^
Pd_2_Cl_6_^2–^	–252
[PdCl_3_(H_2_O)]^−^	–215

The addition of the capping agent to the Pd^2+^ solution in PD led to no substantial changes in the observed spectrum (cyan dotted line vs blue line in Figure 2). Conversely, upon reduction with NaBH_4_ (red line), the band at 418 nm was not evident while an increased background absorption and an increase at short wavelengths with a maximum absorption at 285 nm were observed.

The observed increment in the background intensity can be attributed to the scattering induced by the formation of colloidal palladium particles and could be responsible for the masking/disappearance of the band at 418 nm. The blue shift of the CT band could be correlated to the increase of pH because of the NaBH_4_ addition. However, the exact assignment of the observed band at 285 nm is still undetermined. According to Klasovsky et al., this peak relates to plasmon excitation in the colloidal particles.^69^ However, Boily argued that it is also compatible with a CT transition of Pd chloro–hydroxo complexes, PdCl_*x*_(OH)_*y*_^*n*–^, which are stable solution species under our experimental conditions according to the hydrolysis equilibrium.^72^

The effect of adding the AP capping agent to the sol Pd^2+^ with subsequent metal reduction is shown in Figure 3. As expected, the initial UV–vis spectrum of Pd(II) (blue line) is consistent with that of Figure 1, further pointing out the reproducibility of the experimental procedure. Upon addition of AP (green line), the broad band centered at 418 nm is depleted, and the decrease in intensity of the shoulder at 307 nm is accompanied by a broadening and a shift of the peak to 317 nm. These significant changes are a consequence of the chlorine ligand substitution by the AP capping agent.

**Figure 4:**
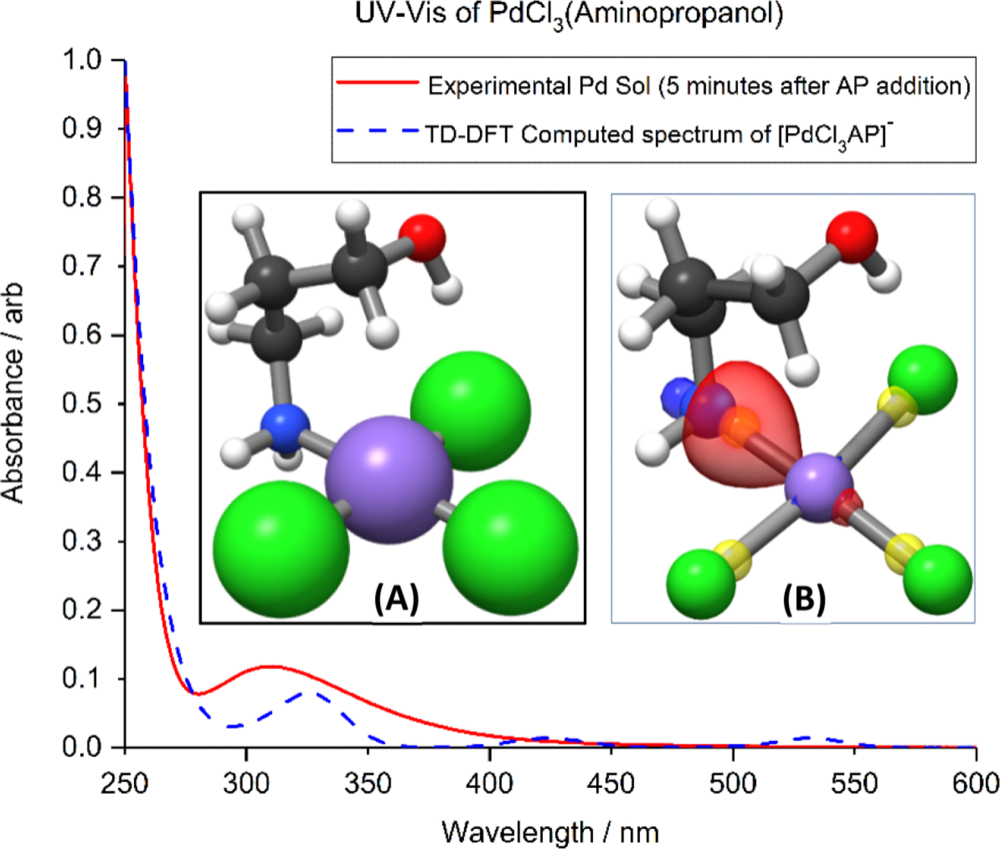
UV–vis spectra of PdCl_4_^2–^ sol in the presence of the capping agent AP (green) and computed TD-DFT spectrum of PdCl_3_AP (blue dashed). Inset: (A), PdCl_3_AP^–^ lowest energy geometry; (B), [PdCl_3_AP]^−^ with the Pd–N localized bonding orbital and the centroids for the Pd–N and Pd–Cl bonds. Color code: Pd: lilac; Cl: green; O: red; N: blue; C: dark gray; H: white; and localized orbital centroids: translucent yellow.

These results are consistent with the computational analysis; the TD-DFT spectrum of the most favorable product, [PdCl_3_AP]^−^, (Figure 4, dashed blue line) was also found to be consistent with the experimental results. The energies of reaction for the formation of aminopropanol ligand complexes from PdCl_4_ are shown in Table 2.

**Table 2:** Energy Change of Reaction for the Potential Products of the Reaction between PdCl_4_ and AP

potential products	Δ*E*_r_/kJ mol^–1^
PdCl_2_OHAP (cis)	–207
PdCl_2_OHAP (trans)	–188
PdCl_3_AP	–305
PdCl_2_AP_2_	–295

The peak observed at 334 nm in the computational spectrum shows an excitation from a hybrid Pd d–Cl p orbital to a molecular orbital composed from the palladium sp and a hybridization of orbitals in the aminopropanol ligand.

This agreement with the experimental spectrum strongly suggests that the aminopropanol is directly attached to a Pd^2+^ species in a structure consistent with the computational predictions. A similar suggestion was reported by Groppo et al.^32^ who compared the diffuse reflectance UV–vis spectra of bulk Pd(OAc)_2_ diluted in SiO_2_ and in pyridine. The bulk Pd(OAc)_2_ in silica exhibited a band maximum at 400 nm, whilst the one diluted in pyridine revealed a peak centered around 330 nm. As a possible explanation, the authors suggested that one or two acetate ligands were substituted using pyridine units.

This interaction between the NH group and the Pd^2+^ ions involves a strong interaction between the electron-rich amino group and the metal ion, which could induce a change in the actual oxidation state of the metal by partial reduction. For this reason, centroid analysis of the Pipek–Mezey localised orbitals was utilized to examine the oxidation state of Pd and the character of the AP–metal bond, Figure 4B. The localized two-centered bonding orbitals indicated that the Pd–AP bond was largely dative in character with the electrons in the bonding orbital being biased toward the more electronegative nitrogen. Analysis of the single atom orbitals showed the electronic configuration of the Pd atom to be consistent with Pd(II), 4s^2^4p^6^4d^8^ because of the presence of eight centroids centered upon the Pd atom and the bonding orbital centroids being biased toward the ligands. Coordination of the ligand does not therefore involve a redox process.

Upon reduction with NaBH_4_, the UV–vis spectrum (Figure 3, red line) assumed a very broad profile, where it is difficult to uniquely identify defined features or any eventual shifts. A significant increase in the background absorbance was indeed observed, which, as in the case of PD, was attributed to Willis–Tyndall scattering which is characteristic for the formation of particles.

**Figure 5:**
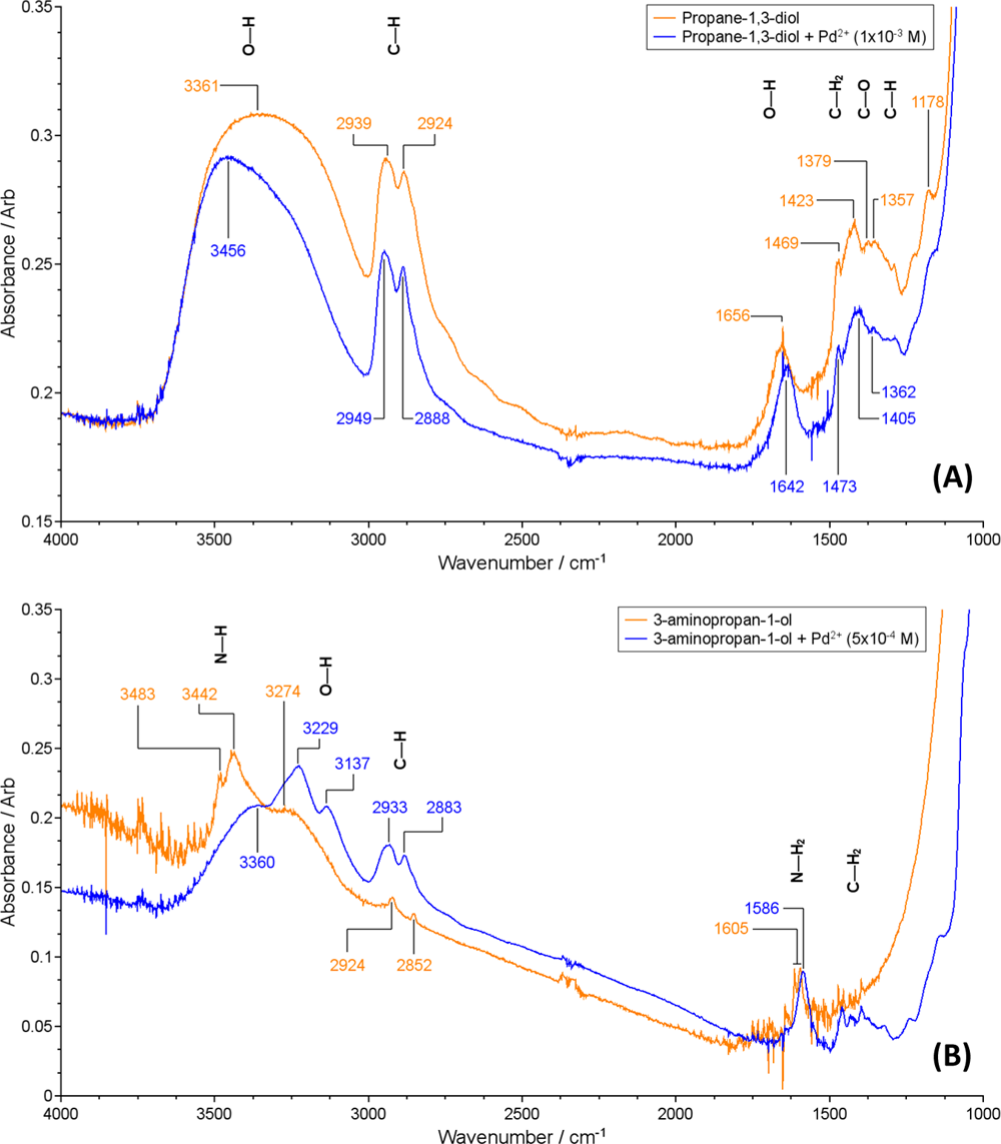
(A) FT-IR spectra of PD and Pd–PD (top). (B) FT-IR spectra of AP and Pd–AP (bottom).

In order to obtain more information on the coordination of PD and AP to Pd^2+^ ions, FT-IR spectra of the Pd(II) complexes were recorded and compared to the FT-IR spectra of the pure ligand molecules, as shown in Figure 5. In the case of propane-1,3-diol, Figure 5A, the interaction with Pd^2+^ ions seems to provoke negligible perturbations in the molecular structure of the ligand. Indeed, in addition to an overall decrease in intensity upon Pd^2+^ addition, only an erosion of the broad peak at 3361 cm^–1^ because of the stretching of −OH groups, together with a shift of the peak related to the −OH bending mode from 1656 to 1647 cm^–1^ are observed, which indicate that the presence of the Pd^2+^ ions perturbed these groups. This spectroscopic feature points out that the interaction between the metal ions and the propane-1,3-diol ligand occurs through the −OH groups.

Conversely, more significant changes were observed in the FT-IR spectrum of AP after interaction with Pd^2+^ ions (Figure 5B). The peaks observed at 3483 and 3442 cm^–1^, related to the symmetric and antisymmetric stretching modes of the −NH group, are decreased and new bands at 3229 and 3137 cm^–1^ are produced immediately upon the addition of Pd^2+^. Moreover, the −N–H bending mode observed at 1605 cm^–1^ is shifted to 1586 cm^–1^. To rationalize these differences, in particular, to understand if the peaks at 3229 and 3137 cm^–1^ are the result of a marked shift of the symmetric and antisymmetric stretching modes of the −NH group or of a strong perturbation of the −OH group, frequency calculations were performed in order to simulate FT-IR spectra for the most stable conformations of the Pd(II)–AP complex.

**Figure 6:**
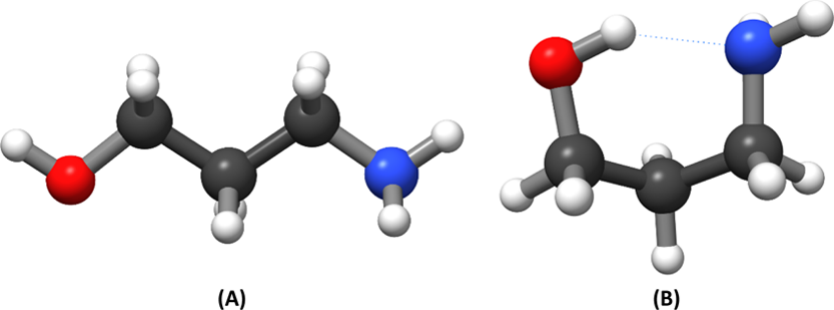
Optimization of two different conformations of the AP adsorbate in the gas phase: (A) all trans, (B) conformation with internal H-bond.

Different configurations of the adsorbate were first optimized in the gas phase in order to determine the most stable configuration. Upon optimization with a plane-wave basis set and the PBE functional, a straight chain configuration and an internally hydrogen bonded conformation (with the hydroxyl oxygen to the amino nitrogen) were found to be the most favorable, with the latter being the most stable. The H-bonded conformation was found to have an electronic energy ≈ 0.8 eV lower than that of the other structure, which was also consistent with results obtained using an atom-centered basis set. The two configurations are shown in Figure 6.

These results confirmed the former hypothesis coming from the FT-IR discussion, indicating that the interaction between the metal ions and the AP ligand occurs through the −NH group. The complete assignments of the FT-IR bands are summarized in Table 3.

**Table 3:** Vibrational Frequencies and Assignments of the FT-IR Bands

vibrational frequency (cm^–1^)	assignment (vibrational mode)
**Bands Observed for PD**	
3361	–OH stretching
2939 and 2924	–CH symm and asymm stretchings
1656	–OH bending
1469	–CH_2_ bending
1423	C–O stretching
1379	C–H bending
**Bands Observed forPd–PD**	
3456	–OH stretching
2949 and 2888	–CH symm and asymm stretchings
1642	–OH bending
1473	–CH_2_ bending
1405	C–O stretching
1362	C–H bending
**Bands Observed for AP**	
3483 and 3442	–NH symm and asymm stretching
3274	–OH stretching
2924 and 2852	–CH symm and asymm stretchings
1605	–OH bending
**Bands Observed forPd–AP**	
3360	–OH stretching
3229 and 3137	–NH symm and asymm stretching
2933 and 2883	–CH symm and asymm stretching
1586	–OH bending

**Figure 7:**
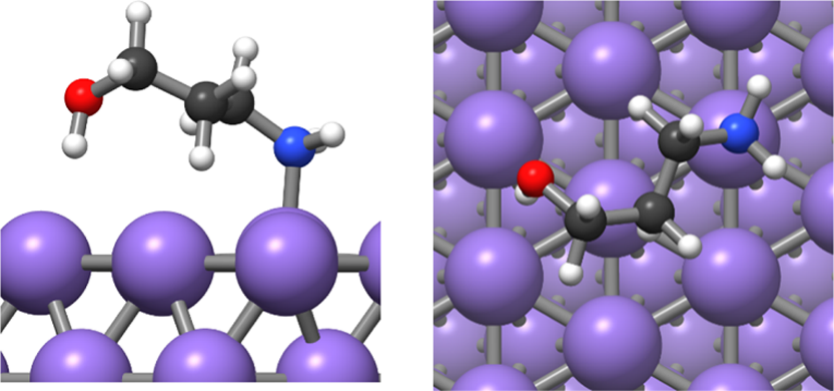
Adsorption of the AP adsorbate on the Pd(111) surface with the nitrogen binding to the surface.

**Figure 8:**
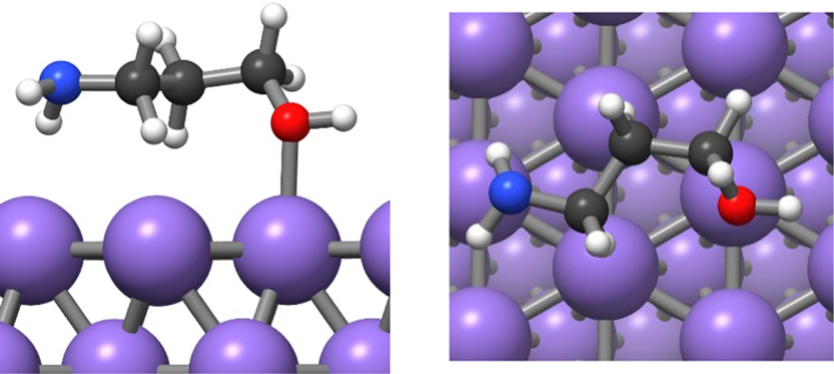
Adsorption of the AP adsorbate on the Pd(111) with the oxygen binding to the surface.

**Figure 9:**
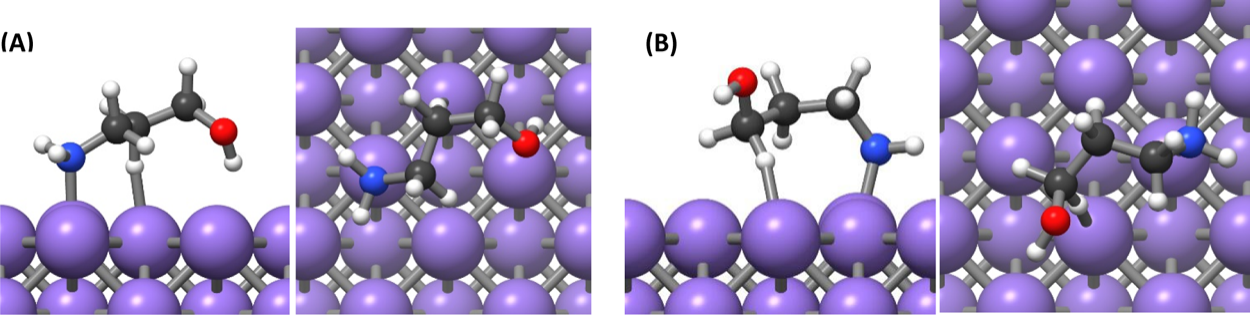
Adsorption of the AP adsorbate on Pd(100) with the nitrogen binding to the surface and (A) oxygen pointing downward and (B) oxygen pointing upward.

**Figure 10:**
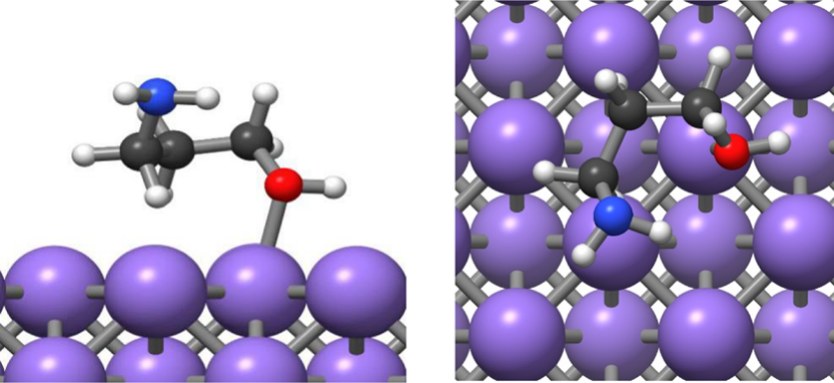
Adsorption of the AP adsorbate on Pd(100) with the oxygen binding to the surface.

For this reason, the interaction of the AP molecule with metallic Pd surfaces was also investigated. The most stable configuration of AP was then adsorbed on the two low index surfaces of palladium metal: Pd(111) and Pd(100). Structures with AP binding to the surface through the oxygen atom or the nitrogen atom were investigated; it was found that those in which both the oxygen and the nitrogen atom bind to the surface at the same time were unstable because of internal strain within the AP. In the case with AP adsorbed through the amine N atom, hydroxyl hydrogen is also attracted toward the surface. Table 4 and Figures 7–10 summarize the results obtained.

**Table 4:** Adsorption Energies of the Different Configurations of 3-Aminopropan-1-ol on Pd(100) and Pd(111) Surfaces

configuration	energy of adsorption/kJ mol^–1^	N–Pd or O–Pd distance/Å
Pd(111) surface		
nitrogen binding	–145	2.15
oxygen binding	–95	2.34
Pd(100) surface		
nitrogen binding (O up)	–127	2.16
nitrogen binding (O down)	–149	2.15
oxygen binding	–86	2.32

The conformation with the nitrogen interacting with Pd was found to be more stable on both Pd(111) and Pd(100) surfaces as was also seen for the molecular precursor. Because the Pd(111) surface is more stable than the Pd(100) surface, the bonding of the adsorbate to the Pd(100) surface is stronger. As a result, the ligand is preferentially adsorbed on Pd(100) facets, making the surface less accessible. The reduced accessibility of Pd(100) facets favors the NP growth on the Pd(111) facet, resulting in a greater surface area of Pd(100) facets. This observation confirms the important role of the capping agent in controlling the growth mechanism and directing the final particle shape. In addition, the selective blocking of the Pd(100) surface can have an important impact on the catalytic performances of Pd NPs.

**Figure 11:**
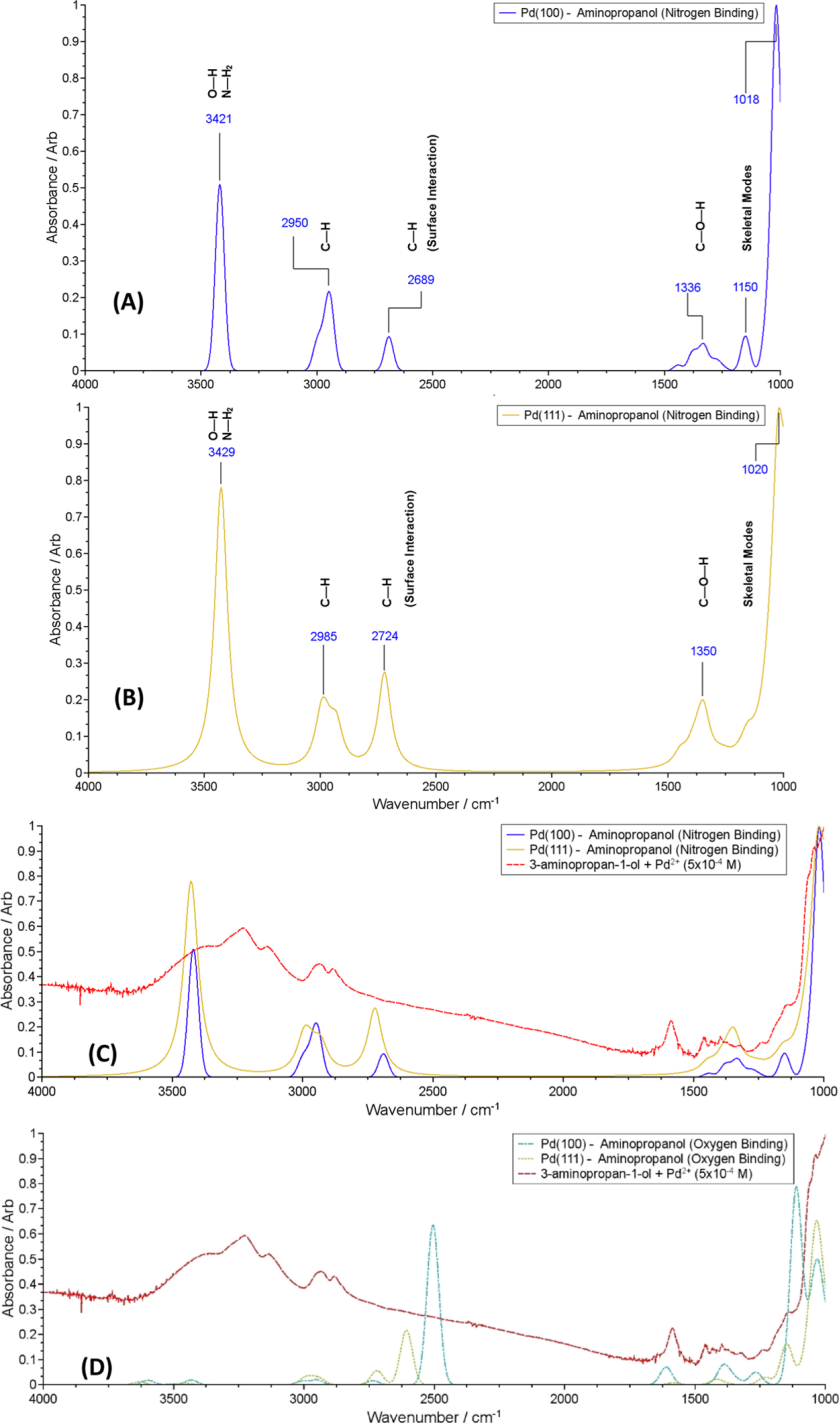
IR spectra for the most stable conformations: (A) nitrogen binding of the aminopropanol on the Pd(100) surface (B) nitrogen binding of the aminopropanol on the Pd(111) surface (C) plots A and B overlaid with the experimentally obtained IR spectrum, (D) oxygen binding on the Pd(111) and Pd(100) surfaces overlaid with the experimentally obtained IR spectrum.

Frequency calculations on the most stable configurations were performed and IR spectra were calculated. The resulting IR spectra for the different conformations are reported in Figure 11. The correlation between the experimental IR spectrum and the spectra obtained for the adsorbed configurations can be taken as a strong indication that the aminopropanol caps the NP surfaces by bonding through the amino moiety. This is further supported by the oxygen binding spectra which indicate that the O–H vibrational modes are significantly less infrared active than the nitrogen binding O–H modes in both the experimental and computed nitrogen binding spectra. While the accuracy of the extended-surface model for many properties of small NPs is limited, the assignment of the spectrum to the N-binding species should not be of concern. The spectra for the nitrogen binding of AP on the Pd(111) and Pd(100) surfaces are very similar. Given that the difference between the two surfaces is the number of surrounding atoms, it can be concluded that the coordination number of Pd has very little effect on the vibration energies. Therefore, if the NPs are small to the extent that the corner or edge sites are significant, the adsorption of AP on these sites is not likely to have a major effect on the resultant spectra.

## 4 Conclusions

The synergistic combination of experimental spectroscopies and DFT modeling has allowed us to determine the coordination of small ligand to Pd species during the MNP formation process. Interesting differences emerged between O- and N-containing ligands. In particular, it seems that AP reacts with the molecular precursors by displacing a chloride ion to form a Pd(II)Cl_3_AP complex. AP is able to direct the growth processes during the subsequent reduction by stabilizing the Pd(100) facets and thus exerting a directing influence upon the shape of the resultant metal NPs. Although these molecules are model systems and greater complexity is expected from the adsorption of macromolecules such as polymers, typically used as capping agents, these results help in understanding the phenomena occurring at the interface between the metal surface and the ligand layer. These data will also provide a stimulus to a deeper investigation on the role of the capping agent in metal NP synthesis and in their catalytic behavior.

## Supporting Information Available

Images of a commercial transmission cell for UV–vis; geometries in *XYZ* of optimized complexes employed in this work; and complementary theory–experiment IR spectrum of [PdCl_3_AP]^2+^ (PDF)
