# Supplementary material for: DFT-Assisted Spectroscopic Studies on the Coordination of Small Ligands to Palladium: From Isolated Ions to Nanoparticles
Source: J Phys Chem C Nanomater Interfaces. 2020 Jan 27;124(8):4781–90. doi: 10.1021/acs.jpcc.9b09791 (PMC8016172; doi:10.1021/acs.jpcc.9b09791)

Supplementary Information of:

DFT-assisted spectroscopic studies on the coordination of small ligands to palladium: from isolated ions to nanoparticles

Sebastiano Campisi,[a] Cameron Beevers,[b] Ali Nasrallah,[b] C. Richard A. Catlow,[b] Carine e. Chan-Thaw,[a] Maela Manzoli, Nikolaos Dimitratos, David J. Willock,[b] Alberto Roldan*,[b] Alberto Villa*,[a]

[a] Dipartimento di Chimica, Università degli Studi di Milano, via Golgi 19, I-20133Milano, Italy

[b] Cardiff Catalysis Institute, School of Chemistry, Cardiff University, Main Building, Park Place, CF10 3AT, Cardiff, United Kingdom.

[c] Department of Drug Science and Technology and NIS - Centre for Nanostructured Interfaces and Surfaces, University of Turin, Via P. Giuria 9, 10125 Turin, Italy.

[d] Dipartimento di Chimica Industriale e dei Materiali, ALMA MATER STUDIORUM Università di Bologna, Viale Risorgimento 4, 40136 Bologna, Italy

This supplementary material contains:

- Images of a commercial transmission cell for UV-Vis.
- Geometries in XYZ of optimised complexes employed in this work.
- A complementary theory-experiment IR spectrum of $[PdCl_3Ap]^{2+}$.

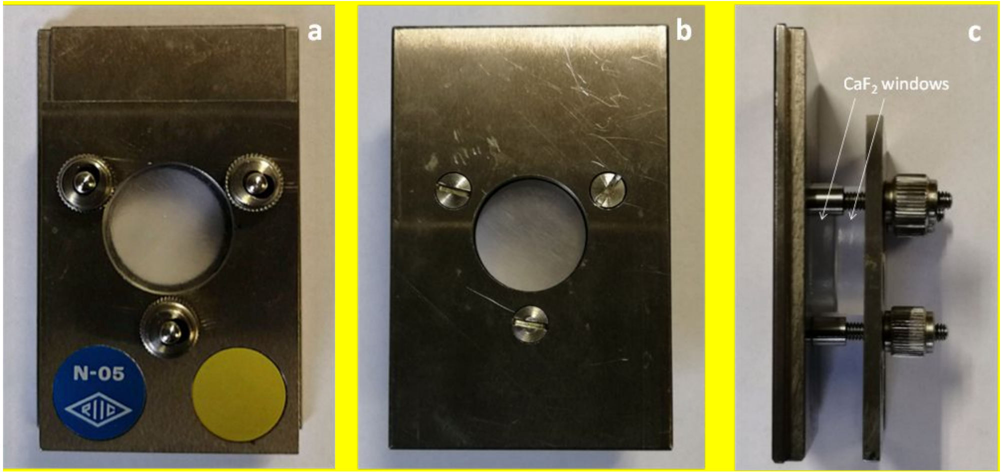

Figure SI-1. Front (a), back (b) and side (c) views of the commercial demountable transmission cell equipped with CaF_2 windows.

Geometries (Å)

$PdCl_4$:

Element	x	y	z
Pd	1.07895346571620	0.09422829828717	0.00000000000000
Cl	3.33523540963971	0.78292558605297	0.00000000000000
Cl	-1.17732312332969	-0.59438506098544	0.00000000000000
Cl	0.39035390993853	2.35054211955262	0.00000000000000
Cl	1.76762033803525	-2.16203094290732	0.00000000000000

Pd_2Cl_6 (Dimer):

Element	x	y	z
Pd	0.67607250845386	-0.12827462548393	0.16914746543272
Cl	2.62005906981037	1.11245188084670	0.00413669744979
Cl	-1.38856437439359	-1.24680075830901	0.36407451231762
Cl	-0.61017993101700	1.80045941459778	0.59051063960201
Cl	1.79741187152819	-2.11046524392405	-0.22950831891358
Pd	-2.67486242453855	0.68196729079124	0.78514283179326

Cl	-4.61971502143675	-0.55812644151641	0.94461301831807
Cl	-3.79524169840652	2.66345848299769	1.18992315400012

$PdCl_3OH_2$:

**Element**	**x**	**y**	**z**
O	-1.50196232045149	-1.12058936707295	-0.03197153026705
Cl	-0.52549693217276	1.82666288644295	0.53767044610278
Pd	-2.51476314318662	0.67426615902901	0.66200167002252
Cl	-4.26512727883487	-0.88220898140810	0.82595477226106
Cl	-3.68343674267179	2.45659098225644	1.40781094270289
H	-2.29027262079364	-1.63389159490379	0.29161016511088
H	-0.81720996188883	-1.17034908434357	0.65752353406693

$PdCl_3$(Aminopropanol) species:

Element	x	y	z
Pd	2.39365098378012	0.02398964701504	2.92715296829366
Cl	2.43458311573323	0.08993464274970	0.63608896153657
Cl	0.51870459405290	1.39891739377137	3.10442680947071
Cl	4.28284796982823	-1.35434019764555	3.04356720435640
H	0.11824466814410	-1.43251532990819	5.49770354321271
C	2.25399641213389	-1.19950148754468	5.80222655674680
H	3.16787616003865	-1.77079053192355	5.59139225006808
H	2.97417712411309	-3.03579484861298	3.64710676704761
C	1.03072242335363	-2.05259307884571	5.47732422622104
O	2.26343880055220	-3.60742511652687	4.02587202394991
C	1.10450169743986	-2.79204411723393	4.13399919021738
H	1.06060715519501	-2.06189826370307	3.30516550526353
H	0.93546355436302	-2.80006102123724	6.28105325436854
H	0.22511511342594	-3.45057987985997	4.04492738886582
N	2.32791322308224	0.05114932968543	5.01252066514952
H	2.25068738746919	-0.94542525101095	6.87676371023927
H	1.50573766828593	0.65247778662087	5.15795535679974
H	3.16461194900889	0.57657032421039	5.27303361819261

Figure SI-2. Infrared spectrum of $[PdCl_3Ap]^{2+}$ with the experimental I.R. spectrum for comparison:

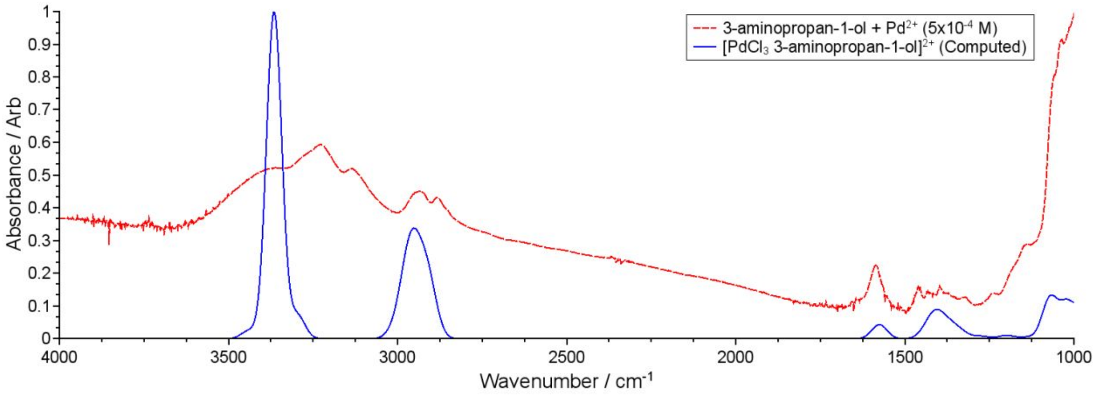

Supplement: Supplementary file 1 — jp9b09791_si_001.pdf [file jp9b09791_si_001.pdf]
